# *Chlorella sorokiniana* Extract Improves Short-Term Memory in Rats

**DOI:** 10.3390/molecules21101311

**Published:** 2016-09-29

**Authors:** Maria Grazia Morgese, Emanuela Mhillaj, Matteo Francavilla, Maria Bove, Lucia Morgano, Paolo Tucci, Luigia Trabace, Stefania Schiavone

**Affiliations:** 1 Department of Clinical and Experimental Medicine, University of Foggia, Foggia 71121, Italy; mariagrazia.morgese@unifg.it (M.G.M.); luciamorgano@alice.it (L.M.); paolo.tucci@unifg.it (P.T.); stefania.schiavone@unifg.it (S.S.); 2 Department of Physiology and Pharmacology, “Sapienza” University of Rome, Rome 00185, Italy; emanuela.mhillaj@uniroma1.it (E.M.); maria.bove@uniroma1.it (M.B.); 3 STAR Agroenergy Research Group, University of Foggia, Via Gramsci, 89-91, Foggia 71121, Italy; matteo.francavilla@unifg.it; 4 Institute of Marine Science, National Research Council, via Pola 4, Lesina 71010, Italy

**Keywords:** *Chlorella sorokiniana*, short-term memory, emotional behaviour, serotonin, noradrenaline, hippocampus

## Abstract

Increasing evidence shows that eukaryotic microalgae and, in particular, the green microalga *Chlorella*, can be used as natural sources to obtain a whole variety of compounds, such as omega (ω)-3 and ω-6 polyunsatured fatty acids (PUFAs). Although either beneficial or toxic effects of *Chlorella sorokiniana* have been mainly attributed to its specific ω-3 and ω-6 PUFAs content, the underlying molecular pathways remain to be elucidated yet. Here, we investigate the effects of an acute oral administration of a lipid extract of *Chlorella sorokiniana*, containing mainly ω-3 and ω-6 PUFAs, on cognitive, emotional and social behaviour in rats, analysing possible underlying neurochemical alterations. Our results showed improved short-term memory in *Chlorella sorokiniana*-treated rats compared to controls, without any differences in exploratory performance, locomotor activity, anxiety profile and depressive-like behaviour. On the other hand, while the social behaviour of *Chlorella sorokiniana*-treated animals was significantly decreased, no effects on aggressivity were observed. Neurochemical investigations showed region-specific effects, consisting in an elevation of noradrenaline (NA) and serotonin (5-HT) content in hippocampus, but not in the prefrontal cortex and striatum. In conclusion, our results point towards a beneficial effect of *Chlorella sorokiniana* extract on short-term memory, but also highlight the need of caution in the use of this natural supplement due to its possible masked toxic effects.

## 1. Introduction

Increasing evidence shows that algae, and in particular eukaryotic microalgae, can be used as natural sources to obtain a number of food and non-food products [1,2]. Moreover, because of the high cost of microalgae production technology, academia and industry are looking for new applications of algae extracts/products that could contribute to increase the economic value of biomass with a “biorefinery” approach [3]. Within the food science and nutrition field, the identification and characterization of new bioactive compounds, which are able to confer additional health benefits beyond the basic nutritional and energetic requirements in order to maintain and promote consumers’ health and prevent chronic diseases is, at present, a hot-topic [2]. Considering the tremendous market value of the functional food industry, valued in 168 billion dollars just in the US in 2010 [4], it is easy to understand the enormous interest in the identification of new compounds, extracts and products that, once their efficacy has been proved with scientific evidence, can be produced at a larger scale. Among them, there is a whole variety of compounds, such as polyunsatured fatty acids (PUFAs), which are widely known to exert pharmacological or nutraceutical effects [5]. In particular, the PUFA composition in many species of the green microalga *Chlorella*, belonging to the phylum *Chlorophyta*, has been well characterized and the correlations between cultivation, conditions and percentages of fatty acids have been the focus of many studies [6]. Among all the *Chlorella* strains, the *Chlorella sorokiniana* has been shown to be the most suitable one to extract omega (ω)-3 and ω-6 PUFAs, mainly by biorefinery-based production methods [7,8,9]. Together with their beneficial effects against cardiovascular system disorders and their protective actions on uncontrolled cellular proliferation, ω-3 PUFAs have been demonstrated to be important physiological components of total brain lipid amount and to play a crucial role in several neurological functions, such as neurogenesis, neurotransmission and protection against oxidative stress-induced cerebral damage [10,11]. Further on, they are precursors of molecules involved in the modulation of cerebral immune and inflammatory processes [12,13]. Reduced dietary ω-3 PUFAs content has been shown to be associated with impaired cognitive and behavioural performance [14] and correlated to the development of depressive and anxiety-like symptoms [15]. In this context, we have recently demonstrated that lifelong nutritional ω-3 PUFAs deficiency evokes depressive-like state through soluble β amyloid (βA) involvement in rats [16]. Accordingly, ω-3 PUFAs have been also demonstrated to have important neuroprotective effects in both rodent models of neurodegenerative diseases [17,18] and human clinical investigations [19,20]. On the other hand, the role of ω-6 PUFA in brain maturation and development is much less studied. It is known that the main ω-6 derivative, arachidonic acid (AA), crucially contributes to several physiological functions, acting both as an important precursor of bioactive mediators and as an activator of protein kinases and ion channels involved in the processes of synaptic transmission [21]. Recently, the involvement of a ω-6 PUFA-enriched diet in enhancing plasma levels of βA has been reported in rodents [16]. In addition, a high ω-6/ω-3 ratio appears to be particularly unfavorable for proper central nervous system functioning [22]. Importantly, *Chlorella sorokiniana* is marketed in the US as a dietary supplement for weight control and protection against cancer, oxidative-stress and age-dependent cognitive impairment, as well as metabolic and cardiovascular diseases [23]. On the other hand, some recent reports have shown side toxic effects following dietary supplementation with *Chlorella sorokiniana*, such as gastrointestinal disorders, allergic reactions and increased skin sensitivity to the sun, elevation in plasma levels of uric acid and, more rarely, tubulointerstitial nephritis and psychotic symptoms development [24,25,26]. Although both the beneficial or toxic effects of *Chlorella sorokiniana* have been attributed to its specific ω-3 and ω-6 PUFAs content [23], the underlying molecular pathways remain to be elucidated yet. The aim of the present study was to investigate the effects of an acute oral administration of a lipid extract, containing mainly ω-3 and ω-6 PUFAs, obtained from monoxenic strain of *Chlorella sorokiniana* on the cognitive, emotional and social behaviour in rats and to identify the possible underlying neurochemical alterations. By using a double experimental approach (extraction and chemical characterization of the total lipid component on one hand and analysis of its activity on behaviour and neurochemistry on the other hand), we demonstrated specific behavioural effects of *Chlorella sorokiniana* lipid extract in rats, together with a specific brain region neuromodulatory activity.

## 2. Results

### 2.1. Chemical Characterization of Chlorella Sorokiniana Extract

The algal biomass showed a total lipids (TL) content of 21% dry weight (d.w.), which was composed mainly by fatty acids (42% d.w.) and unsaponified fraction (UF) (19% d.w.). The remaining 39% d.w. was composed by water-soluble compounds including short polypeptides, glycerol, monomeric sugars and salts. The bioactivity of the TL fraction was characterized in terms of chemical composition of its two main components: unsaturated fatty acids (UFs, Table 1) and fatty acid methyl esters (FAMEs, Table 2).

The most abundant UF compounds were 3,7,11,15-tetramethyl-2-hexadecen-1-ol and ergosterol. As shown in Table 2, FAMEs belonging to the C_16_ and C_18_ carbon chain groups were the most abundant, and accounted for 92% of the total FAMEs detected, according to previously reported results [27,28]. Among them, PUFAs showed a concentration of 286.7 mg·g^−1^ TL, while monounsaturated (MUFA) and saturated (SFA) fatty acids were found in lower concentrations. PUFAs were composed by ω-3 (169.5 mg·g^−1^ TL) and ω-6 (114.6 mg·g^−1^ TL) fatty acids in a ratio of 1 to 1.5 (ω-3 to ω-6). α-Linolenic acid and (*Z,Z,Z*)-7,10,13-hexadecatrienoic acid were the predominant ω-3 fatty acids, while linoleic acid and (*Z*,*Z*)-7,10-hexadecadienoic acid were the most abundant ω-6 fatty acids.

### 2.2. Effects of Acute Oral Administration of Chlorella Extract on Cognition and Short-Term Memory

**Figure 1:**
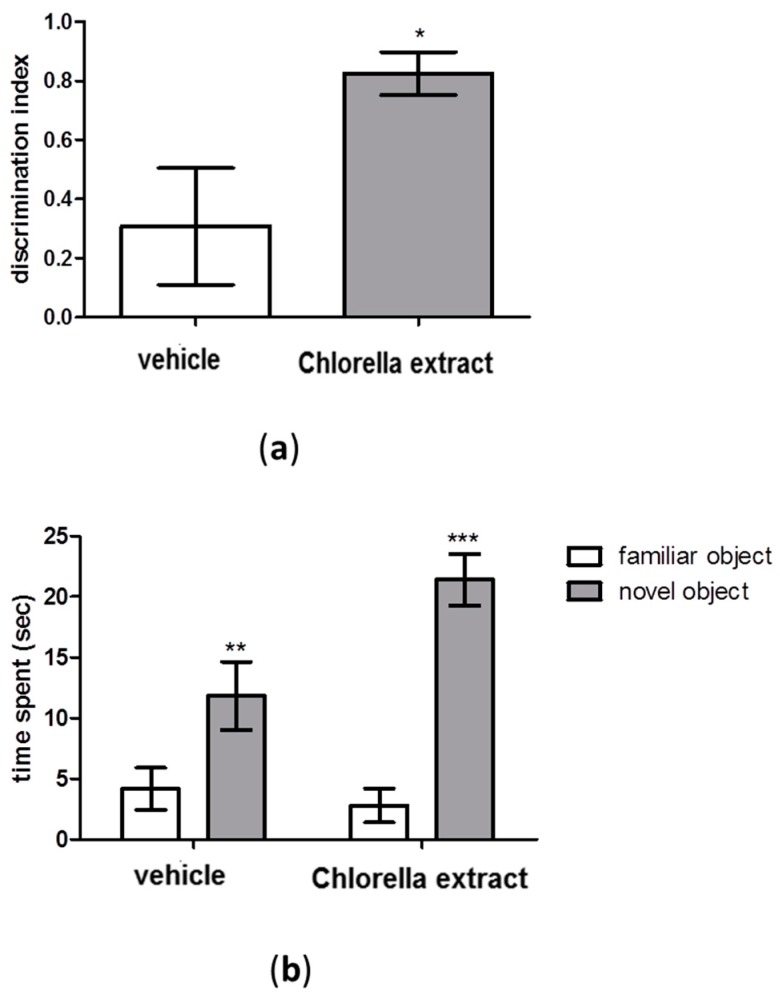
The NOR test in male Wistar rats after *Chlorella sorokiniana* extract or vehicle administration. (**a**) discrimination index and (**b**) time spent in the NOR test of male Wistar rats after *Chlorella sorokiniana* extract (Chlorella extract, 30 mg/kg/mL dark bar) or vehicle (sunflower oil 1 mL/kg, empty bar) administration. Data are expressed as mean ± SEM (*n* = 8 per group); (**a**) *t*-test * *p* < 0.05 vs. vehicle-treated rats; (**b**) Two-way ANOVA followed by Bonferroni’s multiple comparisons test. ** *p* < 0.01 vs. familiar object and *** *p* < 0.001 vs. familiar object for vehicle- and for *Chlorella* *sorokiniana*-treated rats, respectively.

The effects of an acute *Chlorella sorokiniana* administration on cognition and short-term memory were assessed by performing the Novel Object Recognition (NOR) test. Results showed that the discrimination index was significantly increased in *Chlorella sorokiniana*-treated rats compared to controls (Figure 1a, unpaired *t*-test; *p* < 0.05). Moreover, both *Chlorella sorokiniana*- and vehicle-treated rats exhibited the same exploratory performances in the NOR test, with a significant difference in total time spent exploring the new object rather than the familiar one (Figure 1b, Two-way ANOVA; *F*_(1,28)_ = 37.33, *p* < 0.0001, followed by Bonferroni’s multiple comparisons test; familiar vs. novel object; *p* < 0.001 and *p* < 0.01 for *Chlorella* *sorokiniana* extract and vehicle, respectively).

### 2.3. Effects of Acute Oral Administration of Chlorella Sorokiniana Extract on Locomotion

**Figure 2:**
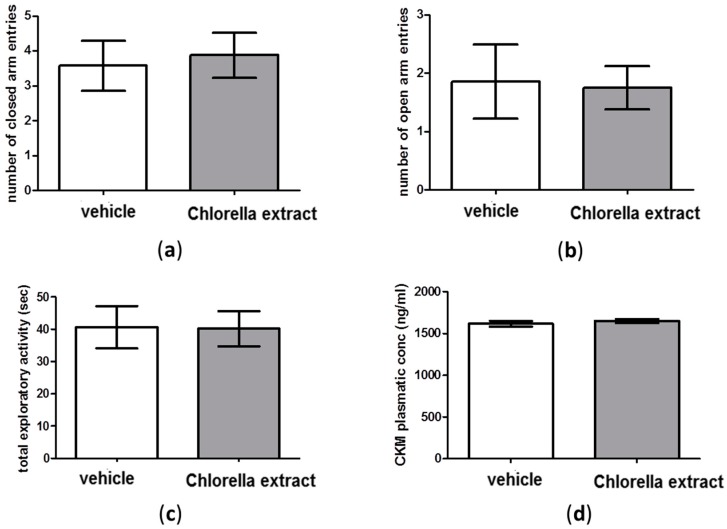
Evaluation of locomotion parameters in male Wistar rats after *Chlorella sorokiniana* extract or vehicle administration. (**a**) Number of closed arms entries and (**b**) number of open arms entries in the elevated plus-maze test; (**c**) total exploratory activity in the novel object recognition test; (**d**) plasma CKM concentrations Wistar rats after *Chlorella sorokiniana* extract (Chlorella extract, 30 mg/kg/mL dark bar) or vehicle (sunflower oil 1 mL/kg, empty bar) administration. Data are expressed as mean ± SEM (*n* = 8 per group).

As shown in Figure 2a,b, there were no significant differences in the number of entries into closed and open arms between *Chlorella sorokiniana*- and vehicle-treated rats in the Elevated Plus Maze (EPM) test (unpaired *t*-test; n.s.). Moreover, as reported in Figure 2c, no statistical differences were found in total exploration time in the NOR test between *Chlorella sorokiniana*- and vehicle-treated rats (unpaired *t*-test; n.s.). Since this lack of effect on locomotion could have been induced by a muscle damage, and since the appearance of creatine-kinase M-type (CKM) in blood has been generally considered to be a marker of skeletal muscle damage, we measured plasma CKM concentrations in *Chlorella sorokiniana*- and vehicle-treated rats. Results showed no difference between experimental groups (unpaired *t*-test; n.s.; Figure 2d), thus confirming that *Chlorella* *sorokiniana* administration did not affect locomotor activity, at least under our experimental conditions.

### 2.4. Effects of Acute Oral Administration of Chlorella sorokiniana Extract on Emotional Profile

**Figure 3:**
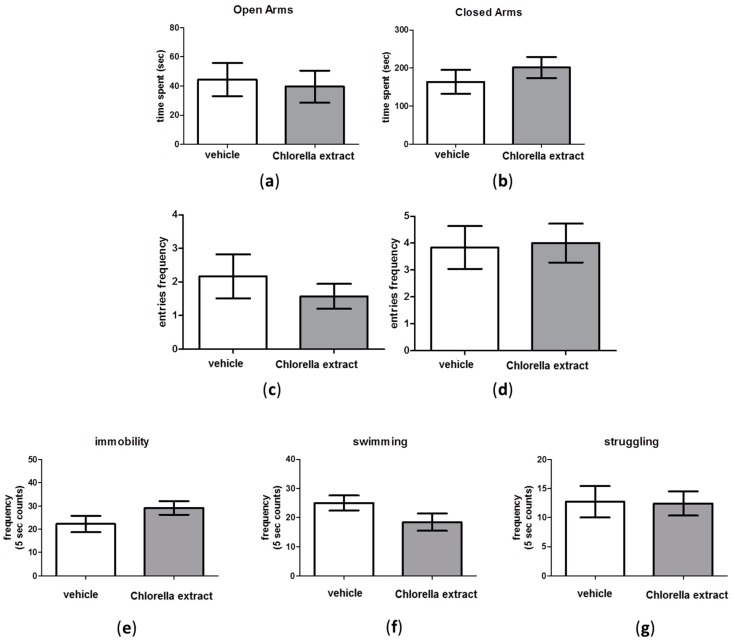
The forced swimming test and elevated plus maze test in male Wistar rats after *Chlorella sorokiniana* extract or vehicle administration. (**a**) Amount of time spent in open and (**b**) closed arms, (**c**) the entries frequency into open and (**d**) closed arms in the EPM test; (**e**) Immobility; (**f**) swimming and (**g**) struggling frequency in the FST. Male Wistar rats were treated with *Chlorella sorokiniana* extract (Chlorella extract, 30 mg/kg/mL dark bar) or vehicle (sunflower oil 1 mL/kg, empty bar) administration. Data are expressed as mean ± SEM (*n* = 8 per group).

We did not detect changes in anxiety- and depressive-like behaviour between *Chlorella sorokiniana*- and vehicle-treated groups in EPM and Forced Swimming Test (FST). Indeed, as shown in Figure 3a–d, no significant differences were found in the time spent into open and closed arms and in the entries frequency into open and closed arms (unpaired *t*-test; n.s.) between experimental groups in the EPM test. Likewise, no differences in the immobility, swimming and struggling frequency in *Chlorella sorokiniana*-treated rats, compared to controls, were reported in the FST (Figure 3e,g unpaired *t*-test; n.s.).

### 2.5. Effects of Acute Oral Administration of Chlorella sorokiniana Extract on Central Monoamine Content

**Figure 4:**
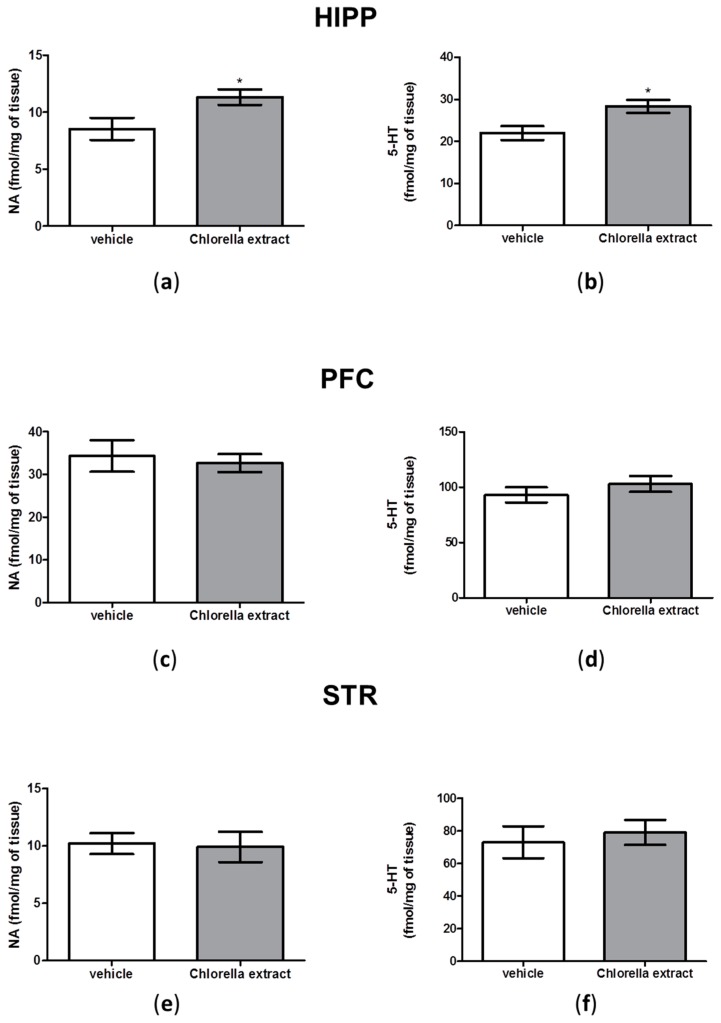
Monoamine quantification in male Wistar rats after *Chlorella sorokiniana* extract or vehicle administration. NA and 5-HT levels in HIPP (**a** and **b**); PFC (**c** and **d**); STR (**e** and **f**) of male Wistar rats after *Chlorella sorokiniana* extract (Chlorella extract, 30 mg/kg/mL dark bar) or vehicle (sunflower oil 1 mL/kg, empty bar) administration. Data are expressed as mean ± SEM (*n* = 8 per group); (**a**) *t*-test * *p* < 0.05 vs. vehicle-treated rats and (**b**) *t*-test * *p* < 0.05 vs. vehicle-treated rats.

In order to corroborate behavioural results with neurochemical data, we measured serotonin (5-HT) and noradrenaline (NA) content in hippocampus (HIPP), prefrontal cortex (PFC) and striatum (STR) of *Chlorella sorokiniana*- and vehicle-treated animals. Hippocampal 5-HT content was significantly increased in *Chlorella sorokiniana*-treated rats compared to controls animals (Figure 4b, unpaired *t*-test; *p* < 0.05). Similarly, we found that hippocampal NA concentrations were significantly higher in animals that received *Chlorella* *sorokiniana* administration compared to controls (Figure 4a, unpaired *t*-test; *p* < 0.05). On the other hand, no differences between *Chlorella sorokiniana*- and vehicle-treated animals were found in PFC and STR with respect to NA and 5-HT contents (Figure 4c–e, unpaired *t*-test; n.s.).

### 2.6. Effects of Acute Oral Administration of Chlorella sorokiniana Exctract on Social Behaviour

**Figure 5:**
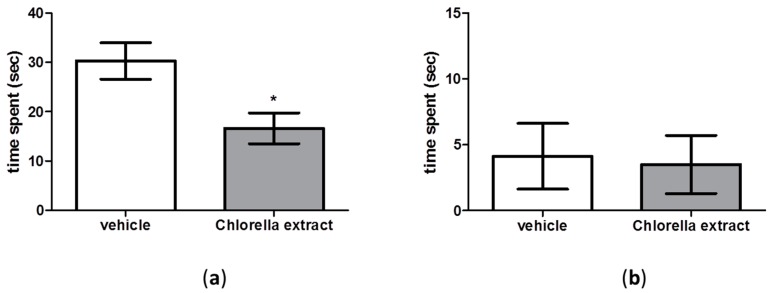
The social interaction test in male Wistar rats after *Chlorella sorokiniana* extract or vehicle administration. (**a**) Time spent performing social and (**b**) time spent performing aggressive behaviour. Male Wistar rats were treated with *Chlorella sorokiniana* extract (Chlorella extract, 30 mg/kg/mL dark bar) or vehicle (sunflower oil 1 mL/kg, empty bar) administration. Data are expressed as mean ± SEM (*n* = 8 per group); (**a**) *t*-test * *p* < 0.05 vs. vehicle-treated rats.

Results showed that social behaviour was significantly decreased in *Chlorella sorokiniana*-treated animals with respect to controls (Figure 5a, unpaired *t*-test; *p* < 0,05), while no differences were found in aggressive behaviour between *Chlorella sorokiniana*- and vehicle-treated rats (Figure 5b, unpaired *t*-test; n.s.).

## 3. Discussion

In the present study we found that an acute oral administration of *Chlorella sorokiniana* lipid extract was able to significantly improve memory performance in rats. This behavioural outcome was corroborated by neurochemical data indicating a significant increase in NA and 5-HT at the hippocampal level.

In previous studies, *Chlorella sorokiniana* was reported to be an attractive source of bioactive compounds, foods, feeds and fuels [27,29,30]. Those findings make *Chlorella sorokiniana* an interesting and challenging biomass that could be used to find new and more valuable bioactive molecules.

Interestingly, we found that the lipidic fraction extracted from *Chlorella sorokiniana*, rich in PUFA of either ω-3 and ω-6 families, induced in rats an increased interest in exploring a novel object in respect to a previously encountered familiar one. This test represents a simple and reproducible behavioural assay primarily based on innate and spontaneous exploratory behaviour in rodent without the use of any reinforcement paradigm or stressful stimuli. It has been demonstrated that the NOR test allows one to study either short or long term memory, depending on the behavioural paradigm chosen [31]. In our experimental conditions, by using an intertrial interval of 1 minute, we tested the effect of treatment on short-term memory. We found for the first time that the lipidic fraction of *Chlorella sorokiniana*, particularly rich in linoleic and α linolenic acids (ALA), can improve memory function after a single oral administration. In searching of putative targets, peroxisome proliferator-activated receptors (PPAR) can be suitable candidates. Indeed, linoleic acid and linolenic acids have been reported to directly bind PPARα and γ receptors [23]. Activation of these intracellular receptors has been shown to improve memory and cognition in several animal models, either after global cerebral ischemia [32], or in diabetes-induced cognitive dysfunction [33], or in transgenic mice model of mental retardation [34].

In particular, activation of PPARγ receptors at a hippocampal level was shown to improve brain derived neurotrophic factor (BDNF) expression [33], and to rescue the long-term potentiation (LTP) induction by restoring α-amino-3-hydroxy-5-methyl-4-isoxazolepropionic acid (AMPA) receptor expression and extracellular signal–regulated kinases-cAMP response element-binding protein (ERK-CREB) activities [34]. In further agreement, transient transfection of constitutively active PPARγ plasmid in hippocampal neuronal cells was reported to be able to increase BDNF, AMPA, and *N*-methyl-d-aspartate (NMDA) receptors expression and spine formation [34]. On the other hand, ALA, the most concentrated fatty acids in *Chlorella* *sorokiniana* extract, was hypothesized to modulate NMDA functioning and BDNF release by influencing membrane fluidity [35]. Indeed, ALA can markedly increase membrane fluidity that correspond to a better efficiency in formation of lipid rafts in neuronal membranes [36]. Lipid raft composition, and thus lipid micro-environments on the cell surface, by favouring specific protein-protein interactions can influence the activation of signaling cascades [37]. Such an enhanced efficiency of transmembrane signaling would result in increased activated NMDA receptors, which are part of lipid raft [38]. This event would lead to higher calcium influx and initiation of signal transduction pathways ending up to activation of nuclear factor kappa B (NF-κB) via the canonical pathway which then translocates to the nucleus, ultimately leading to increased BDNF mRNA and protein levels [39,40,41]. Enhanced intracellular BDNF would result in its higher secretion, thereby binding to its receptors Tropomyosin receptor kinase B (TrkB), and thus controlling synaptic function [42]. Our results are in line with the findings of other studies reporting that short term supplementation with ω-3 PUFA can improve brain cognitive function by altering α-synuclein, calmodulin and transthyretin genes expression in the HIPP [43]. It has been shown that α-synuclein can regulate the homeostasis of monoamines in synapses, through modulatory interactions of the protein with monoaminergic transporters, particularly for NA [44,45]. The NA transporter (NET) is the primary mechanism of NA reuptake and termination of noradrenergic transmission [46,47]. Thus, it can be speculated that PUFA present in our *Chlorella* *sorokiniana* fraction, by altering α-synuclein, may modulate NET efficiency leading to increased NA levels. Indeed, in our experimental conditions, we found a significant increase in NA content only at hippocampal level. Otherwise, modulation of plasmalemma fluidity and lipid raft composition could explain such neurochemical alteration. Indeed, as previously hypothesized, increased NMDA receptor signaling may explain the increased NA release, considering that in rat hippocampal synaptosome preparations, NMDA agonists were shown to enhance NA release [48]. On the other hand, it should be taken into account that increased neurotransmitter contents can also reflect a reduction in their release or an increase in their storage. In this regard, very few data are available concerning the effects of PUFA rich extract on 5-HT and NA increased storage. Reduction in dopamine vesicle density in the rat frontal cortex has been reported after ω-3 PUFA deficiency [49], although a mechanism involving an alteration in vesicle density would appear hardly applicable in our experimental conditions considering the short overlap between drug exposure and time of measurement. In any case, we cannot completely rule out such hypothesis and further studies are necessary to elucidate this possible mechanism.

Our neurochemical result endorses the behavioural outcome on short-term memory. Indeed, we found increased NA and 5-HT content only in the HIPP, a brain area important for both spatial memory and non-spatial objects or items experienced, as a form of event memory [50,51,52]. It was reported that NA controls multiple brain functions, such as attention, perception, arousal, sleep, learning, and memory. The HIPP is innervated by noradrenergic projections and hippocampal neurons express β-adrenergic receptors (β-AR). These receptors modulate long-lasting forms of synaptic potentiation, such as LTP, a neuronal process crucially involved in long-term storage of spatial and contextual memories [53]. Furthermore NA, by acting on hippocampal β-AR, was shown to regulate spatial memories and in particular the content and persistence of synaptic information storage in the hippocampal subfields. In addition, activation of these receptors is graded according to the novelty or saliency of the experience [54]. Likewise, it has been shown that stimulation of adenylate cyclase following β-AR activation is the putative mechanism through which NA produces LTP in the dentate gyrus of the HIPP [55].

On the other hand, 5-HT was also increased after *Chlorella* *sorokiniana* administration. Serotonergic neurotransmission is responsible for regulating hippocampal plasticity and electrical activity, as well as hippocampal-dependent behaviours and cognitive performance [56]. In particular, it has been shown that by acting at 5-HT_1A_ receptors at hippocampal level, increased 5-HT levels can improve memory performance [57]. Thus, we can hypothesize that the increase in 5-HT content in the HIPP of treated rats could neurochemically underline the improvement of short-term memory induced by *Chlorella* *sorokiniana* lipidic fraction.

On the other hand, considering the short duration of the exposure to algal fraction we cannot exclude that any central neurochemical alterations found actually reflect peripheral activation. In this regard, PPAR are highly expressed in epithelial cells of colon and, to a lesser degree, in macrophages and lymphocytes [58]. Furthermore, peripheral release of hormones after food intake has been associated to beneficial effect on memory formation [59].

Ultimately, it has been demonstrated that gut microbiota is actually able to metabolize PUFA and derived metabolites can accumulate in plasma of treated mice [60]. In this light, alteration in cognitive function have been found after gut microbial perturbation while memory enhancement was described after probiotic treatment (see [61] for a review). Therefore, further studies aimed at elucidating possible peripheral effects at the gut level are warranted.

On the other hand, we evaluated the emotional profile in rats after *Chlorella sorokiniana* administration and no alterations in depressive-like or anxiety-like behaviours were retrieved, as revealed by FST and EPM, respectively, although a different strain of *Chlorella*, *Chlorella vulgaris*, has been proposed as adjuvant therapy in depressed and anxious medicated patients [62]. However, discrepancies can be justified on the basis of the different algal strains, algal harvesting and culture biorefinery processes and thus different fatty acid compositions. It is well known that impaired serotonergic neurotransmission in the PFC area is central to depressive disorders [63] and increases in cortical 5-HT and NA have been related to higher swimming and struggling frequencies, respectively, in the FST [64,65]. Thus, the absence of neurochemical alterations in PFC are in line with the absence of alterations found in the FST outcomes. In addition, our behavioural outcomes were validated by the fact that no locomotion alterations were found after *Chlorella* *sorokiniana* extract administration, as revealed by total exploration time in NOR test, CKM plasma levels and full entries in EPM.

Finally, we found that *Chlorella sorokiniana* administration, while it did not affect aggressive behaviour as indicated by a lack of alteration in noradrenergic content in PFC [66], it reduced the social behaviour. This behavioural paradigm can be used to study sociability [67]. Previous studies have negatively correlated fronto-cortical 5-HT metabolism with this behavioural trait [67], although conflicting results have been reported [68,69]. On the other hand, increase in 5-HT release at a hippocampal level has been linked to decreased social behaviour in rats, but according to the authors, such neurochemical modifications cannot be considered the only explanation [70]. Accordingly, in our conditions, while no alterations in PFC were noted, a significant increase was found in serotonergic hippocampal content, possibly explaining the behavioural outcome. However, it should be noted that differences in methodological procedures, such as sample collection and processing, timing after last behavioural testing, and rat strains could account for some of the variance observed across the literature, thus future studies in this direction are surely warranted in order to evaluate the safety profile of our algal extract.

In conclusion, we have described for the first time the beneficial effect of lipidic fraction of *Chlorella sorokiniana* on short-term memory after acute administration. *Chlorella sorokiniana* is marketed in the US as a dietary supplement for age-dependent cognitive impairment [23] and is generally considered safe. Although our study supports this evidence, our data also indicate that attention should be paid to the use of this natural supplement for its possible masked toxic effects.

## 4. Experimental Section

### 4.1. Microalgae Cultivation

Monoxenic strain of *Chlorella sorokiniana* (UTEX 2805) was phototrophically cultivated in an outdoor closed vertical tubular photobioreactor (PBR) (400 L volume) at the STAR*Facility Centre of the University of Foggia (Foggia, Italy), and grown in Bold Basal medium during the period from April 2015 and June 2015, under a solar radiation (PAR) that ranged between 700 and 930 W·m^−2^. Flow rate of air was 10 L·W·min^−1^, while CO_2_ was injected on pH demand (pH_set point_ = 8.00). The temperature in the PBR ranged between 18 and 25 °C. All the parameters were monitored online by SCADA Software (Version 2.0.1, Aqualgae SL, Vigo, Spain). The algal growth rate was monitored each day by measuring the optical absorbance at 550 nm. The PBR operated in a semi-continuous way with a dilution rate of 0.25 day^−1^ and a mean productivity of 25.12 g m^−2^·day^−1^. The harvested culture was daily centrifuged using a semi-continuous centrifuge (Westfalia, GEA Westfalia Separator Group GmbH, Oelde, Germany). The dewatered algal biomass was then freeze-dried and stored at −20 °C.

### 4.2. Extract and PUFA Characterization

Lipids from freeze-dried algal biomass were extracted as previously reported by Francavilla et al. [71]. TL were saponified and the saponified mixture was then transferred into a separatory funnel, and the Teflon tube was washed with 40 mL of Milli-Q (Millipore, Milan, Italy) water. The unsaponified matter in the combined solution was then extracted four times with 20 mL of n-hexane. The hexane phases were combined, dried overnight with sodium sulphate, filtered and evaporated. The UF was analysed by means of gas chromatography-mass spectrometry (GC-MS, IT-240, Agilent Technologies, Santa Clara, CA, USA), as previously described by Francavilla et al. [72]. Hydroalcoholic residue of extraction process of UF was acidified until pH 1 by adding HCl 2 M and extracted three times with 25 mL of *n*-hexane. The hexane phases were combined, dried with sodium sulphate anhydrous, filtered and evaporated. The yellowish viscous liquid obtained (fatty acids) was treated with 10 mL of freshly prepared 5% methanolic HCl in a Teflon tube for microwave. This mixture was carefully mixed and the tube was closed under nitrogen and then heated for 20 min in a microwave oven (MARS 6, CEM Corporation, Buckingham, UK) at 80 °C. After cooling to room temperature, 5 mL of 6% aqueous K_2_CO_3_ were added and the mixture was extracted four times with 20 mL of *n*-hexane. The hexane phases were then combined, dried with sodium sulphate anhydrous, filtered and evaporated. The process yielded a yellowish viscous liquid (FAMEs) that was weighed and stored at −20 °C until GC-MS analysis. FAMEs were analysed by GC-MS/MS equipment at 110 °C for 5 min, followed by a 2 °C·min^−1^ ramp up to 260 °C, followed by 5 min at 260 °C. The injector temperature was 250 °C and the injected volume was 1 µL. FAME peaks were identified by comparison of their retention times and mass spectra with those of a standard mixture (PUFA C4-C24, Supelco, Bellafonte, PA, USA), whereas they were quantified by using calibration curves made with PUFA C4-C24 standard (Supelco) and using C15:0 FAME (Supelco) as internal standard.

### 4.3. Animals and Treatment

Adult male Wistar rats (Harlan, S. Pietro al Natisone, Udine, Italy) weighing 250–300 g were used in this study. They were housed at constant room temperature (22 ± 1 °C) and relative humidity (55% ± 5%) under a 12 h light/dark cycle with ad libitum access to food and water. Procedures involving animals and their care were conducted in conformity with the institutional guidelines of the Italian Ministry of Health (D.L. 26/2014), the Guide for the Care and Use of Mammals in Neuroscience and Behavioural Research (National Research Council 2004), the Directive 2010/63/EU of the European Parliament and of the Council of 22 September 2010 on the protection of animals used for scientific purposes and to ARRIVE guidelines. “3Rs” were pursuit in every experimental procedure. Animal welfare was daily monitored through the entire period of experimental procedures. No signs of distress were evidenced and all efforts were made to minimize the number of animals used and their suffering.

Two experimental groups were considered: control animals treated with sunflower oil as vehicle and rats administered the *Chlorella* *sorokiniana* extract by gavage at the dose of 30 mg/kg diluted in 1 mL of sunflower oil. Behavioral and neurochemical experiments were performed 40 min after oral administration of *Chlorella* *sorokiniana* extract. Behavioral and neurochemical protocols were carried out in separate subset of animals in order to avoid confounding factors derived by carry over effects.

### 4.4. NOR Test

The NOR test was performed according to Giustino at al. [73]. Briefly, rats were submitted to two habituation sessions where they were allowed for 5 min to explore the apparatus (circular arena, 75 cm diameter). 24 h after the last habituation, a session of two 3-min trials, separated by a 1-min intertrial interval (retention interval), was carried out. The discrimination index was calculated using the following formula: (N − F) / (N + F) (N = times spent in exploration of the novel object during the choice trial; F = times spent in exploration of the familiar object in the choice trial) [74].

### 4.5. FST

The FST was performed according to Porsolt et al. [75]. On the first test day, animals were placed individually in inescapable Perspex cylinders (diameter 23 cm; height 70 cm), filled with a constant maintained 25 ± 1 °C temperature water at a height of 30 cm [65]. During the pre-conditioning period, animals were observed for 15 min. Then, rats were removed and dried before to be returned to their home cages. Twenty-four hours later, each rat was positioned on the water-filled cylinder for 5 min. This session was video-recorded and subsequently scored by an observer blind to the treatment groups. During the test sessions, the time spent in performing the following behaviours was measured: struggling (time spent in tentative of escaping), swimming (time spent moving around the cylinder) and immobility (time spent remaining afloat making only the necessary movements to keep its head above the water).

### 4.6. Social Interaction Test

The social interaction procedure was adapted from File et al. [76], as previously described [77]. The test was performed in a circular open arena (made of dark plastic; diameter 60 cm; height 31 cm), unfamiliar to the animals and placed in a deemed lit room. On the day of testing, all rats were weighed, and pairs were assigned on the basis of weight and treatment. More specifically, *Chlorella sorokiniana*-treated rats were tested with vehicle-treated partners on the basis of weight, ensuring that they did not differ by more than 10 g. The animals were marked on their back and placed head to head simultaneously in the arena, and their behaviour was recorded for 10 min by a camera mounted vertically above the test arena. During quantification, the observer, who was blinded to the experimental conditions, scored the total time that each rat spent performing the following behaviours: sniffing (sniffing several body parts of the other rat, including the anogenital region), following (moving towards and following behind the other rat around the arena), climbing (climbing over and under the conspecific’s back), kicking and boxing. The defined social interactions included the following: sniffing, following and climbing the partner as social behaviour and boxing and kicking as aggressive behaviour parameters.

### 4.7. EPM Test

The EPM test was performed according to Pellow et al. [77]. The apparatus consisted of two opposite Plexiglas open arms (50 cm × 10 cm) without side walls and two closed arms (50 cm × 10 cm × 40 cm) extending horizontally at right angles from a central area (10 cm × 10 cm). The maze was situated in a separate brightly lit room illuminated with four, 32-W fluorescent overhead lights that produced consistent illumination within the room. The apparatus had similar levels of illumination on both the open and closed arms. The maze was elevated to a height of 50 cm in the lit room. At the beginning of the experiment, the rat was placed in the central platform facing an open arm and allowed to explore the maze for 5 min. The following parameters were analyzed: number and frequency of entries into open and closed arms and the time spent in open and closed arms. An arm entry was counted when the hind paws were placed on the open arm.

### 4.8. Post-Mortem Tissue Analysis

Rats were euthanized and brains were immediately removed and cooled on ice for dissection of HIPP, PFC and STR regions, according to the atlas of Paxinos and Watson [78]. Tissues were frozen and stored at −80 °C until analysis was performed.

#### 4.8.1. Monoamine Quantification

5-HT and NA concentrations were determined by high performance liquid chromatography (HPLC) coupled with an electrochemical detector (Ultimate ECD, Dionex Scientific, Milan, Italy), as previously described [16]. Separation was performed by using a LC18 reverse phase column (Kinetex, 150 mm × 3 mm, ODS 5 μm; Phenomenex, Castel Maggiore, Bologna, Italy). The detection was accomplished by a thin-layer amperometric cell (Dionex, Thermo Scientific, Milan, Italy) with a 5 mm diameter glassy carbon electrode at a working potential of 0.400 V vs. Pd. The mobile phase used was 75 mM NaH_2_PO_4_, 1.7 mM octane sulfonic acid, 0.3 mM EDTA, acetonitrile 10%, in distilled water, buffered at pH 3.0. The flow rate was maintained by an isocratic pump (LC-10 AD, Shimadzu, Kyoto, Japan) at 0.6 mL/min. Data were acquired and integrated using Chromeleon software (version 6.80, Dionex, San Donato Milanese, Italy).

#### 4.8.2. Measurement of Plasma Levels of Creatine-Kinase M-Type

Measurement of plasma levels of CKM was performed by using an enzyme-linked immunosorbent assay (ELISA) kit provided by Abcam (Cambridge, UK). Assays were performed according to the manufacturer’s instructions. Each sample analysis was performed in duplicate to avoid inter-assay variations.

### 4.9. Blindness of the Study

All analyses were performed blind with respect to the treatment delivery. The blinding of the data was maintained until the analysis and the collection of data was terminated.

### 4.10. Statistical Analysis

All statistical analysis were performed using Graph Pad 5.0 for Windows (GraphPad Software, La Jolla, CA, USA). All data were tested for normality by performing the Bartlett Test. Data were analyzed by unpaired Student’s *t*-test or two-way analysis of variance (ANOVA), followed by Bonferroni’s *post-hoc* test. Data were expressed as mean ± standard error of mean (SEM). Differences were considered significant only when *p*-values were less than 0.05.

## Figures and Tables

**Table 1:** UFs of the TL fraction extracted from *Chlorella sorokiniana*.

Compound	UF (mg·g^−1^ TL)
10-Heneicosene (c,t)	
2-Dexyl-1-decanol	3.24
3,7,11,15-Tetramethyl-2-hexadecen-1-ol acetate (isomer)	0.43
3,7,11,15-Tetramethyl-2-hexadecen-1-ol acetate (isomer)	0.22
3,7,11,15-Tetramethyl-2-hexadecen-1-ol acetate (isomer)	0.32
3,7,11,15-Tetramethyl-2-hexadecen-1-ol	64.26
Squalene	1.23
Not Identified	8.55
α-Tocopherol	2.72
(22*E*)-Ergosta-5,7,9(11),22-tetraen-3-ol	6.23
(3β,22*E*)-Ergosta-5,8,22-trien-3-ol	9.16
Ergosterol	43.36
(3β)-Ergosta-5,8-dien-3-ol	7.12
(3β,5α)-Ergost-7-en-3-ol	21.56

**Table 2:** FAMEs of the TL fraction extracted from *Chlorella sorokiniana*.

Compound	FAMEs (mg·g^−1^ TL)
Omega 3	169.53
(*Z*,*Z*,*Z*)-7,10,13-Hexadecatrienoic acid methyl ester	69.24
α-Linolenic acid methyl ester	100.29
Omega 6	116.66
(*Z*,*Z*)-7,10-Hexadecadienoic acid methyl ester	35.56
(*Z*,*Z*)-9,12-Heptadecadienoic acid methyl ester	2.06
Linoleic acid methyl ester	79.04
Saturated	58.60
Dodecanoic acid methyl ester	0.02
Tridecanoic acid methyl ester	0.02
Tetradecanoic acid methyl ester	1.27
Methyl 13-methyltetradecanoate	0.35
Pentadecanoic acid methyl ester	0.60
Hexadecanoic acid methyl ester	43.88
15-Methylhexadecanoic acid methyl ester	1.68
Heptadecanoic acid methyl ester	1.84
Methyl stearate	6.17
Eicosanoic acid methyl ester	1.53
Heneicosanoic acid methyl ester	0.16
Docosanoic acid methyl ester	0.36
Tricosanoic acid methyl ester	0.07
Tetracosanoic acid methyl ester	0.15
Pentacosanoic acid methyl ester	0.12
Hexacosanoic acid methyl ester	0.38
Monounsaturated	54.29
Methyl myristoleate	0.25
*cis*-7-Tetradecenoic acid methyl ester	0.72
(*Z*-13-Methyltetradec-9-enoic acid methyl ester	0.37
Methyl palmitoleate	20.50
(*Z*)-7-Hexadecenoic acid methyl ester	2.03
(*E*)-9-Hexadecenoic acid methyl ester	2.88
(*Z*)-9-Heptadecenoic acid methyl ester	2.95
Oleic acid methyl ester	24.47
Methyl 9-eicosenoate	0.13
